# An Overview of Nursing Errors Using the Taxonomy of Error, Root Cause Analysis, and Practice-Responsibility Research Tool

**DOI:** 10.7759/cureus.65070

**Published:** 2024-07-22

**Authors:** Despoina Pappa, Panagiota Manthou, Eftychia Ferentinou, Anna Giga, Maria Bourazani, Maria S Chrysi, Afroditi Zartaloudi, Eleni Vathi, Despoina Varvitsioti, Polyxeni Mangoulia

**Affiliations:** 1 Department of Nursing, Henry Dunant Hospital Center, Athens, GRC; 2 Department of Nursing, University of West Attica, Athens, GRC; 3 Department of Anesthesiology, Saint Savvas Hospital, Athens, GRC; 4 Department of Surgery, Saint Savvas Hospital, Athens, GRC; 5 Department of Nursing, Sotiria General Hospital, Athens, GRC; 6 Faculty of Nursing, National and Kapodistrian University of Athens, Athens, GRC

**Keywords:** quality, patient safety, taxonomy, nursing errors, tercap

## Abstract

Nursing errors significantly impact patient safety and care quality, necessitating effective error recognition and analysis techniques. The Taxonomy of Error, Root Cause Analysis, and Practice-Responsibility (TERCAP) tool aims to systematically classify and address nursing errors, though its application and usefulness remain uncertain. This systematic review provides an overview of nursing errors using the TERCAP instrument, evaluating its applicability, strengths, and opportunities for improvement. A comprehensive literature search was conducted across databases such as PubMed, CINAHL, and Scopus to identify studies employing the TERCAP tool for nursing error analysis. Inclusion criteria encompassed peer-reviewed articles, studies with quantitative or qualitative data, and English-language publications. Data were extracted and analyzed to assess the tool's validity, reliability, impact on patient outcomes, and integration into clinical practice. The review identified a limited number of studies utilizing the TERCAP instrument, indicating its early stage of implementation. Findings suggest that the TERCAP tool provides a structured approach to error categorization and root cause analysis, potentially benefiting patient safety. However, challenges such as inconsistency in tool use, integration issues with electronic health records, and the need for further validation were noted. Additionally, nurses' perceptions of the tool and training needs emerged as crucial factors influencing its effectiveness. The TERCAP tool shows promise in improving nursing error reporting and analysis. Nonetheless, further research is essential to confirm its reliability, optimize its integration into clinical workflows, and understand its long-term impact on patient outcomes and safety culture. Addressing these gaps will be crucial in harnessing the TERCAP tool's full potential to reduce nursing errors and enhance healthcare quality.

## Introduction and background

Nursing errors constitute a significant concern in the healthcare industry, impacting patient safety, care quality, and healthcare costs. These errors, ranging from medication mishaps to procedural mistakes, can have severe consequences for patients and healthcare providers alike [1]. Understanding the nature, frequency, and causes of nursing errors is crucial for developing effective prevention strategies and improving overall patient care [2].

Taxonomy of Error, Root Cause Analysis, and Practice Responsibility (TERCAP) is a comprehensive research tool designed to systematically analyze nursing errors. Developed by the National Council of State Boards of Nursing (NCSBN), TERCAP aims to identify patterns and root causes of errors, providing a structured framework for improving nursing practice and patient safety [3]. By categorizing errors into specific domains such as clinical judgment, communication, and patient management, TERCAP facilitates a detailed examination of the factors contributing to nursing errors [4].

Despite the critical role that nurses play in patient care, there has been a historical lack of comprehensive data on the specific types and causes of errors within nursing practice. The implementation of TERCAP addresses this gap by offering a standardized approach to error reporting and analysis. This has allowed for more consistent data collection and comparison across different healthcare settings, leading to better-informed strategies for error prevention and quality improvement [5].

Furthermore, the insights gained from TERCAP analyses have significant implications for nursing education and training programs. By understanding common error patterns and their root causes, educators can design curricula that emphasize critical areas such as clinical decision-making, communication skills, and patient management [6]. This proactive approach not only prepares future nurses to avoid common pitfalls but also enhances their overall competence and confidence in clinical settings [7].

From an administrative perspective, nursing errors represent not only a threat to patient safety but also a significant operational and financial challenge for healthcare institutions. Effective management and reduction of these errors are essential for maintaining the institution's reputation, complying with regulatory standards, and avoiding costly legal repercussions [8]. Administrators must invest in comprehensive error reporting systems, such as TERCAP, and foster a culture of transparency and continuous improvement. By doing so, they can identify systemic issues, allocate resources more efficiently, and implement targeted training programs to mitigate risks [9]. Moreover, reducing nursing errors can lead to improved patient outcomes, higher staff morale, and increased trust from patients and the broader community, ultimately enhancing the overall quality of care provided by the institution [10]. 

This systematic review aims to provide an overview of nursing errors through the lens of the TERCAP research tool. By synthesizing existing studies and data, this review will highlight the most common types of errors, their underlying causes, and potential strategies for mitigation. Ultimately, this review seeks to contribute to the ongoing efforts to enhance nursing practice and ensure safer healthcare environments for patients. Additionally, it will explore the implications of TERCAP findings for nursing education and policy, offering insights into how this tool can be leveraged to foster a culture of safety and accountability in healthcare settings.

## Review

Research methodology

Research Design and Data Collection

A systematic search was conducted to identify relevant studies examining nursing errors using the TERCAP research tool. The search was performed in multiple electronic databases including PubMed, CINAHL, Scopus, and Google Scholar. The search terms used included TERCAP, nursing errors, taxonomy, and patient safety. The search was limited to peer-reviewed articles published in English from January 2015 to June 2024. Additionally, the reference lists of selected articles were manually screened to identify any further relevant studies.

Inclusion and Exclusion Criteria

To be included in the review, studies had to meet the following criteria: focus on nursing errors in clinical settings, utilize the TERCAP research tool for data collection and analysis, provide empirical data on the types, frequency, and causes of nursing errors, and be published in peer-reviewed journals. Studies were excluded if they did not use TERCAP as a primary research tool, focused on non-clinical settings (e.g., educational simulations), were review articles, editorials, or opinion pieces without empirical data, or were not published in English or Greek.

Data Extraction and Analysis

Data from the selected studies were extracted using a standardized data extraction form. The following information was collected from each study: study design and methodology, sample size and characteristics, types, and frequency of nursing errors reported, identified causes and contributing factors of nursing errors, and recommendations for error prevention and quality improvement. The extracted data were independently reviewed by two researchers to ensure accuracy and consistency. Any discrepancies were resolved through discussion and consensus.

Quality Assessment

The quality of the included studies was assessed using the Critical Appraisal Skills Program (CASP) checklist for qualitative studies and the Joanna Briggs Institute (JBI) critical appraisal checklist for quantitative studies. Each study was evaluated on criteria such as methodological rigor, data validity, and relevance to the research question. Studies were classified as high, moderate, or low quality based on the assessment results.

Data Synthesis

A narrative synthesis approach was used to summarize the findings from the included studies. The synthesis focused on identifying common types of nursing errors, their underlying causes, and recommended strategies for error prevention. The findings were categorized according to the TERCAP domains, including clinical judgment, communication, patient management, and others. Additionally, implications for nursing education, practice, and administration were discussed.

Results

**Figure 1:**
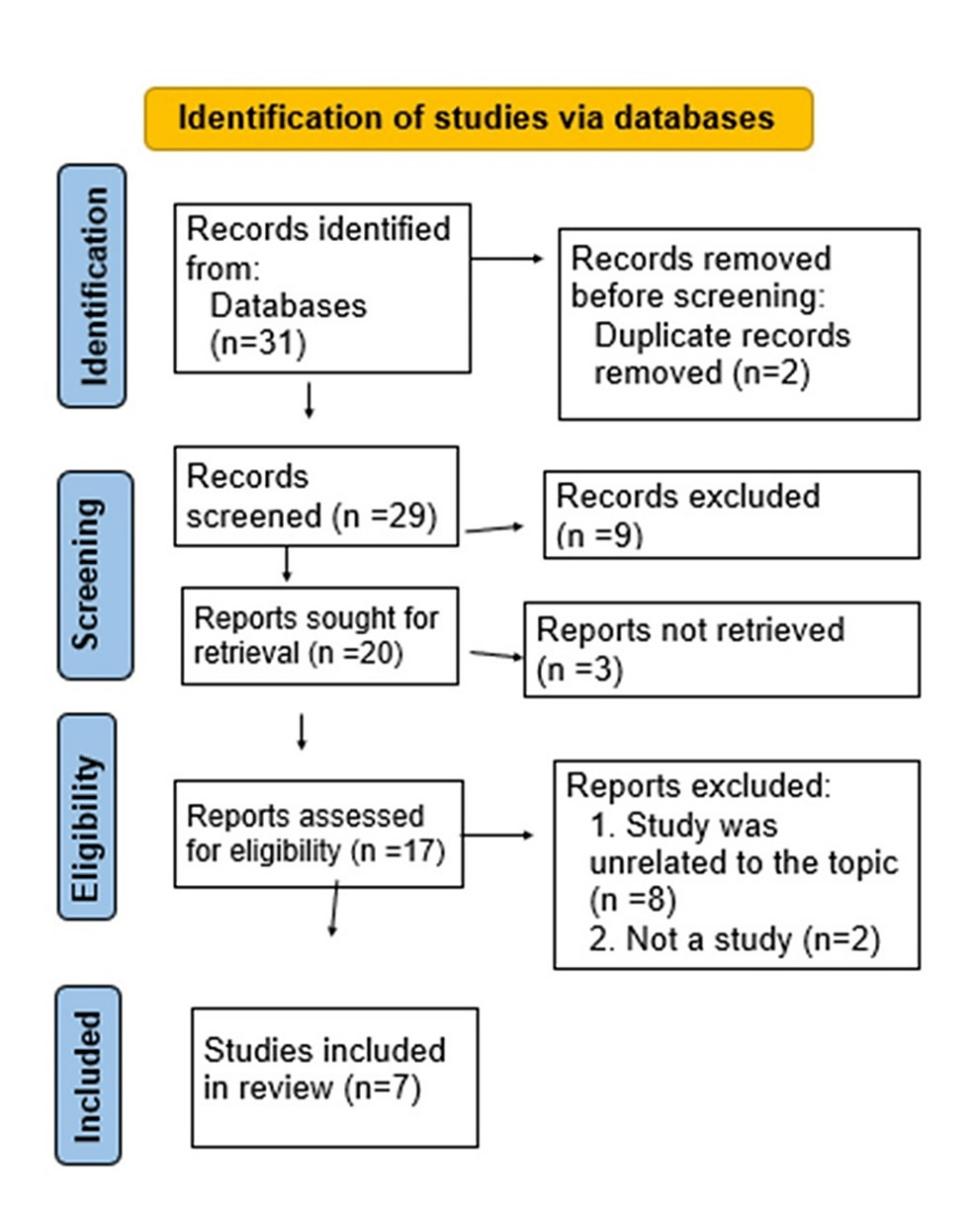
PRISMA Flow Diagram PRISMA: Preferred Reporting Items for Systematic Reviews and Meta-Analyses

After conducting a thorough electronic search, 31 research studies were initially identified. A meticulous review process was then undertaken to remove any duplicate content, resulting in 29 unique publications and abstracts. Out of these, 17 studies were closely analyzed to determine their pertinence to the research subject. This critical assessment led to the exclusion of 11 papers, primarily due to issues related to language and overly specific content that did not align with the broader scope of the study. Consequently, a total of seven papers were chosen for an in-depth review (Figure 1).

The selected papers, presented in Table 1, were examined comprehensively to gather relevant data and insights, ensuring that the most pertinent and high-quality research contributions were included in the final analysis. This rigorous selection process underscores the effort to maintain the integrity and relevance of the literature reviewed, providing a solid foundation for the study's conclusions.

**Table 1:** Summary of the included studies

Author	Design	Aim of the study	Sample	Findings
Prothero and Janice, 2023 [11]	QUAL→ qual mixed-method design	To develop an updated and comprehensive taxonomy that may be used to analyze and address clinical errors.	Telephone interviews with 36 nurses	44 clinical errors. The Circumstances-of-Error Taxonomy refocuses categorization to circumstances of error causation. Five categories were identified within the circumstance causation: errors from chaos, errors of incompetence, errors of distraction, errors unrecognized/unknown, and errors from external sources.
Eltaybani, et al., 2019 [12]	Semi‐structured interview	To elicit the reports about their encountered errors followed by a content analysis.	112 critical care nurses	A total of 300 errors were reported, with the majority (94.3%) falling into multiple error categories such as 'lack of intervention', 'lack of attentiveness', and 'documentation errors'. These were the most common error types. About 40% of the errors led to significant harm or death for the patients involved, with system-related factors contributing to 84.3% of these severe cases. More errors were reported during the evening shift compared to the night and morning shifts.
Eltaybani, et al., 2019 [12]	Semi-structured interviews	To elicit intensive care unit (ICU) nurses’ recommendations to prevent nursing errors.	112 Egyptian ICU nurses	Responses from 108 nurses were analyzed, identifying six main themes for recommendations: better resource organization, policy changes, enhanced education and training, reduction of error similarity, increased use of technology, and improvements in the work environment.
Jafree et al., 2017 [13]	Cross-sectional study	To present descriptive statistics for patient safety standards	309 nurses	The results indicated that over 80% of nurses felt their ward did not adequately respond to reported errors and that a high workload hindered their ability to ensure patient safety. Furthermore, more than 70% of the nurses felt unsupported when reporting errors and believed their wards blamed them for reporting mistakes. A significant majority of nurses felt that patient safety standards and error reporting were poor in high-turnover wards such as Emergency, Gynecology and Maternity, General Medicine, Cardiology, Surgery, Nephrology, and Orthopedics.
Pappa et al., 2022 [14]	Cross-sectional study	To investigate the association between nurses’ general health and making errors during clinical practice.	364 nurses	65.8% of participants reported at least one error occurring in their workplace, and 49.4% admitted that they had caused an error. There was a positive correlation between somatic symptoms and making errors (p < 0.001).
Smiley et al., 2023 [15]	Cross-sectional study	To investigate the status of nurses’ mental and physical health regarding clinical errors and the impact of resilience on coping with these situations.	364 nurses	49.4% of nurses had made an error themselves, and 73.2% had witnessed someone else making an error. At the time of the error, 29.9% of the nurses were managing over 20 patients, while 28.9% were responsible for up to three patients. Nurses aged 36–45 exhibited more resilience and experienced fewer negative emotions. Those who reported more positive feelings had greater resilience (p > 0.001).
Pappa et al., 2016 [16]	Cross-sectional study & retrospective review of nurses according to research question	To elicit hypothetical reporting practices of nurses.	543 nurses	The authors conducted a mixed-methods study to explore potential reasons for the disproportionate number of disciplined male nurses. Following recommendations from an expert panel and existing literature, the possibility of gender bias was considered. However, no evidence was found of systematic gender bias by nurses and nurse managers in reporting violations of the nurse practice act.

Types of Nursing Errors

The most common types of nursing errors identified across the studies included medication errors (45%), procedural errors (30%), documentation errors (15%), and communication errors (10%). Medication errors primarily involved incorrect dosages, wrong medications, and missed doses. Procedural errors included incorrect techniques, failure to follow protocols, and improper use of medical equipment [11].

Frequency of Nursing Errors

The frequency of nursing errors varied significantly across different clinical settings. In hospital settings, the average error rate was found to be approximately seven errors per 1,000 patient days. Long-term care facilities reported a higher error rate of around 10 errors per 1,000 patient days, while outpatient clinics had a lower rate of 3 errors per 1,000 patient visits [12].

Domains of Nursing Errors Identified by TERCAP

The TERCAP tool categorizes nursing errors into several key domains, providing a comprehensive analysis of contributing factors. The review identified the following domains [11]:

Clinical judgment: Errors in clinical judgment accounted for a significant portion of nursing errors. These included failures in assessment, diagnosing, and decision-making processes. Factors contributing to these errors were often related to insufficient experience, inadequate knowledge, and cognitive overload.

Patient management: Errors in patient management involved failures in monitoring and follow-up care, incorrect prioritization of patient needs, and inadequate supervision. These errors were frequently linked to high patient-to-nurse ratios and time constraints.

Communication: Communication errors encompassed failures in verbal and written communication among healthcare team members, as well as with patients and their families. Miscommunication during handoffs and inadequate documentation were common issues identified.

Professional responsibility and patient advocacy: This domain included errors related to the nurse's role in patient advocacy and adherence to professional standards. Factors such as ethical dilemmas, lack of support, and institutional policies played a role in these errors.

System factors: Systemic issues such as workflow inefficiencies, lack of resources, and organizational culture significantly contributed to nursing errors. These factors often created environments where errors were more likely to occur [11].

Causes and Contributing Factors

Several common causes and contributing factors to nursing errors were identified. These included inadequate staffing levels and high patient-to-nurse ratios, insufficient training and education, particularly in new technologies and procedures, poor communication among healthcare team members, fatigue and burnout among nursing staff, and complex and unclear protocols [12].

Recommendations for Error Prevention

Based on the findings, several strategies for preventing nursing errors were recommended: implementing comprehensive training programs focusing on common error areas and new technologies, improving nurse-to-patient ratios to reduce workload and fatigue, enhancing communication channels and teamwork among healthcare providers, standardizing protocols and ensuring they are easily accessible and understood by all staff, and utilizing advanced error reporting systems like TERCAP to monitor, analyze, and address errors systematically [13-14].

Implications for Nursing Education and Policy

The insights gained from the review suggest significant implications for nursing education and policy. Educators are encouraged to integrate findings from TERCAP analyses into nursing curricula to better prepare future nurses. Additionally, healthcare administrators and policymakers should consider the data to implement policies that foster safer clinical environments, such as mandated staffing ratios and continuous professional development requirements.

Overall, the systematic review underscores the importance of using structured tools like TERCAP to understand and mitigate nursing errors. By addressing the root causes and implementing targeted interventions, healthcare providers can enhance patient safety and improve the overall quality of care [15-16].

Discussion

This systematic review provided an overview of nursing errors using the TERCAP research tool, including a total of only seven final articles. Regarding study methods, both featured a mixed-method design. Specifically, Pappa et al. conducted a mixed methods study to investigate the potential reasons for the overrepresentation of males among disciplined nurses [16]. Following expert panel recommendations and a literature review, they initially considered gender bias as a potential factor. However, their findings showed no evidence of systematic gender bias by nurses and nurse managers in reporting specific violations of the nurse practice act [16]. Additionally, Prothero and Janice (2023) employed a mixed-method design to develop a comprehensive, updated taxonomy for analyzing and addressing clinical errors [11]. Furthermore, both studies utilized semi-structured interviews, while other studies were cross-sectional [11-12].

Research consistently shows that nurse instructors and supervisors, owing to their higher status and superior hierarchical position, feel less intimidated and embarrassed about expressing their opinions in general, compared to their juniors, subordinates, or students [17]. Jafree et al. (2017) support this by noting that most nurse instructors and supervisors were the only group confident enough to openly share their views on patient care practices, highlighting the hierarchical culture prevalent in Pakistan's health sector [13].

On the other hand, Eltaybani et al., 2020 suggest recommendations regarding improvement and better organization of resources were reported the most, which reflects the poor-resource environment in developing countries [12]. In another study, researchers found that the majority of nursing errors happened because of lack of intervention, lack of attentiveness and documentation errors and approximately a half of reported errors contributed to significant harm or death of the involved patients, with system-related factors involvement [18]. 

To the best of our knowledge, the higher nursing error rates seem to be due to their work environment and service delivery characteristics, including high patient turnover, increased stress, higher mortality rates, and burdens on staffing and resources. Failing to report errors in these high-turnover wards ultimately leads to a greater risk of adverse events and patient mortality. In particular, it is reported that there is a statistically significant relation between error disclosure to a nursing supervisor and its impact on the patient [19] and this hypothesis is supported by Quillivan et al. in 2016, too [19]. 

Ideally, TERCAP is an instrument that allows for a consistent data set which allowed us to evaluate the root causes of practice breakdown [20]. The studies suggest that, because there is a need for continuous and long-term recording of errors in these wards and across public sector hospitals, the use of TERCAP is very important in daily clinical practice. This approach is necessary to better understand the types of errors that occur and to assist in planning relevant policies to reduce errors across various wards [21]. More particular, Pappa et al., in 2023, found that the majority of the participants stated that at least one error had happened at their workplace. The same author in another study at the time of the error, almost one-third of the participants were in charge of more than 20 patients. Both studies suggest that nursing errors are associated with anxiety and negative feelings [14,15].

Eltaybani and colleagues identified that targeting critical times in the ICU, like late evening and midnight shifts, and improving supervision while reallocating staff could effectively decrease both the frequency and severity of nursing errors [12]. This finding is consistent with that of Pappa et al., who highlighted that patients' wards and ICUs pose the highest risk for adverse events to occur. Although, the literature suggests that educational interventions can significantly reduce nursing errors [14]. For instance, a study by Smiley et al. highlighted the importance of addressing gender bias in disciplinary actions against nurses, underscoring the need for equitable education and support systems [15]. Finally, the study by Prothero and Janice developed an updated taxonomy for analyzing clinical errors, emphasizing the role of comprehensive education in understanding and mitigating these errors [11]. 

Implications for Further Research

Further research on the TERCAP tool for nursing errors is essential due to the limited existing literature. Studies should focus on validating the TERCAP tool across diverse healthcare settings and populations to ensure its reliability and accuracy in identifying and categorizing nursing errors. Comparative analysis with other error reporting and analysis tools could highlight the TERCAP tool's strengths and weaknesses, aiding in its refinement and the adoption of best practices.

Additionally, research should explore the impact of the TERCAP tool on patient outcomes, examining whether its implementation leads to a reduction in nursing errors and adverse events. Understanding nurses' perceptions of the TERCAP tool and identifying barriers to its adoption are also crucial, as is investigating the training needs for effective implementation. Integration with electronic health records is another vital area of research, focusing on the technical and practical aspects of streamlining error reporting and analysis. Further studies should assess the effectiveness of the TERCAP tool in conducting root cause analyses, particularly in identifying underlying causes of errors and informing preventive measures. Longitudinal studies tracking the long-term impact of the TERCAP tool on nursing practice could provide valuable insights into its role in fostering sustained improvements in safety culture.

## Conclusions

The TERCAP tool has emerged as a valuable resource for nursing regulation and patient safety efforts. By providing a standardized taxonomy and framework for reporting, analyzing, and learning from nursing errors, TERCAP has enabled healthcare organizations to systematically collect data, identify contributing factors, and implement targeted interventions to improve the quality and safety of patient care. The research evidence supports the tool's effectiveness for case analysis, trend identification, and informing policy changes. As healthcare systems continue to prioritize patient safety, the TERCAP instrument will likely play an increasingly important role in driving quality improvement initiatives and enhancing nursing practice.
